# Highly efficient and nearly roll-off–free electrofluorescent devices via multiple sensitizations

**DOI:** 10.1126/sciadv.abp9203

**Published:** 2022-07-27

**Authors:** Chen Yin, Yuewei Zhang, Tianyu Huang, Ziyang Liu, Lian Duan, Dongdong Zhang

**Affiliations:** ^1^ Key Laboratory of Organic Optoelectronics, Department of Chemistry, Tsinghua University, Beijing 100084, China.; ^2^ Center for Flexible Electronics Technology, Tsinghua University, Beijing 100084, China.

## Abstract

The efficiency roll-off at high luminance has hindered the wide application of organic light-emitting diodes (OLEDs) for decades. To circumvent this issue, both high exciton utilization and short exciton residence should be satisfied, which, however, faces formidable challenges. Here, we propose an advanced approach of phosphor-assisted thermally activated delayed fluorophor (TADF)–sensitized fluorescence, abbreviated as TPSF. It is proved to be a rational strategy that can realize high quantum efficiency and elaborately accelerated radiative exciton consumption simultaneously by breaking singlet-triplet spin-flip cycles on a TADF host via multiple sensitizations. On the basis of a TADF molecule exhibiting anti–accumulation-caused quenching character, a proof-of-concept device exhibits a maximum external quantum efficiency (EQE_max_) of 24.2% with an ultrahigh *L*_90%_ (the luminance at which EQE drops to 90% of its maximum value) of 190,500 cd m^−2^ and a greatly improved operational stability, unlocking the full potential of OLEDs for ultrahigh-luminance applications.

## Teaser

OLEDs free of efficiency roll-off are implemented by phosphor-assisted TADF-sensitized fluorescence.

## INTRODUCTION

**Fig. 1.**
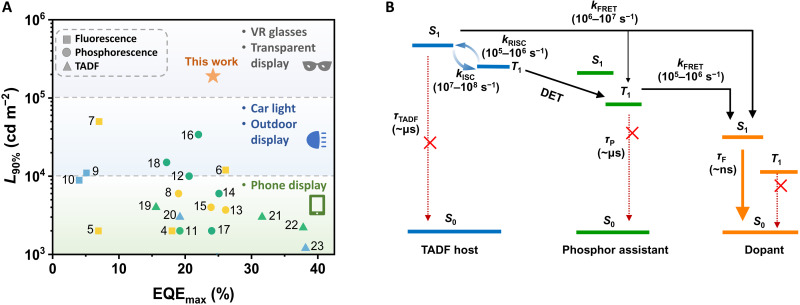
OLED performance summary and energy transfer model. (**A**) Summary of *L*_90%_ as a function of EQE_max_ for reported OLED devices with different emitters. (**B**) Diagram of the exciton process in the TPSF system.

Organic light-emitting diodes (OLEDs) are electroluminescence devices in which excitons formed under electrical excitation can be converted into light by virtue of organic semiconductors (*1*). With the conceptual advancement of both organic emitting materials and device structures, the OLED technology has reached a certain level of maturity with regard to efficiency and stability, particularly under relatively low luminance (~10^3^ cd m^−2^) (*2*). The mobile phone display based on OLEDs with typical luminance levels in this segment have made a successful leap from laboratory to large-scale industrial production. However, commercial uses of OLEDs have been hardly realized for those application scenarios that require extremely high luminance such as general illuminations (~10^4^ cd m^−2^), virtual reality equipment, and even transparent displays (~10^5^ cd m^−2^). It is intrinsically related to a general effect in most OLED structures in which the device efficiency tends to decrease with the increasing luminance or current density, the so-called efficiency “roll-off” (*3*). For practical applications, the luminance is more substantial than the current density. Figure 1 and table S1 summarize the maximum external quantum efficiency (EQE_max_) as a function of the critical luminance *L*_90%_ (namely, the luminance at which EQE drops to 90% of its maximum value) of reported devices with different emitters (*4*–*23*). A clear exclusion of high efficiency and high luminance can be observed, let alone the fact that the 10-fold increase in luminance usually leads to an approximately hundred-fold reduction in device lifetimes. Accordingly, improving device performance at high luminance will most likely realize the further success of OLEDs beyond conventional display applications.

Although the underlying physics is rather complex, various bimolecular annihilation processes have been acknowledged as the dominant reasons for efficiency roll-off under high luminance (*24*). Briefly, those bimolecular annihilation processes strongly depend on both concentrations and residence time of excitons. The former is externally determined while the latter is usually an intrinsic property of emitters. Numerous efforts have been devoted to reduce exciton concentrations by widening the recombination zone to alleviate efficiency roll-off, involving the utilization of bipolar hosts, among which exciplex-forming hosts are the most representative (*25*). Nevertheless, the intrinsic residence lifetimes of emitter-excited states still have a remarkable impact on the roll-off behaviors. Phosphorescent and thermally activated delayed fluorescence (TADF) emitters have exhibited the enormous advantage of recycling triplet excitons for high efficiency (*26*–*31*), but with a rather slow dynamic process (approximately microseconds to milliseconds). Thus, the long-lived triplet excitons will accumulate under high luminance for annihilation and result in a significant efficiency roll-off. The fluorescent dopants, in contrast, can rapidly consume singlet excitons for emission in a nanosecond (~ns) regime and, hence, benefit to suppress exciton annihilation. Recently, by using a scavenger to consume triplet excitons, Adachi and colleagues further suppressed the potential singlet-triplet annihilation in fluorescent OLEDs for alleviated efficiency roll-off (*32*). However, unexpectedly, inconsistent with the common idea that fluorescent OLEDs generally outperform others at a high current density, it is observed in Fig. 1A that the average *L*_90%_ values are relatively similar for both fluorescent and phosphorescent materials, typically in the 100 to 10,000 cd m^−2^ range, with few exceeding this range. The reason should arise from the significantly lower current efficiencies of fluorescent devices, and much higher current densities are required to achieve the same luminance as phosphorescent devices. Therefore, to obtain an ultrahigh *L*_90%_, both the high exciton utilization efficiency and the short residence time of excited states should be satisfied simultaneously. This goal, however, still faces formidable challenges and is yet to be realized since the dawn of OLEDs.

Here, with the aim of high-efficiency and roll-off–free OLEDs, we propose an advanced concept of phosphor-assisted TADF-sensitized fluorescence, abbreviated as TPSF, adopting a TADF-sensitizing host with an anti–aggregation-caused quenching (anti-ACQ) character. Both highly efficient exciton utilization and accelerated exciton consumption were realized in this system by breaking singlet-triplet spin-flip cycles of the TADF host via multiple sensitization processes. In addition, the anti-ACQ property of the TADF host also favors the suppression of host-exciton–involved annihilations. The proof-of-concept OLEDs exhibited a high EQE_max_ with a significantly small roll-off. A remarkable *L*_90%_ of 190,500 cd m^−2^ was observed with a notable *LT85* (the life span at which the device luminance decays to 85% of the initial value under a fixed current density) of 13,215 hours at an initial luminance of 1000 cd m^−2^. This work paves a promising way toward the applications of OLED in practical fields where an ultrahigh-luminance is required.

## RESULTS

### Theoretical analysis of TPSF process

To obtain an ultrahigh *L*_90%_, fast radiative consumption of triplet excitons formed under electrical excitation is the key, which, however, is rather difficult for individual phosphorescence or TADF dopants as previously reported. Sensitized emission using a conventional fluorescent dopant as the final emitter and TADF or phosphorescent materials as sensitizers may provide viability to solve this challenge (*33*, *34*). Generally, fluorescent dopants have a faster radiative rate (*k*_r_) than that of TADF or phosphor, which can potentially accelerate the overall consumption of all electrically generated excitons. In those systems, sensitizers assist the harvest of triplet excitons for high exciton utilization efficiency and subsequently transfer the energy to dopant singlet for radiative decay. Hence, the long-range Förster energy transfer (FRET) from sensitizers to dopants plays an important role in sensitizations. Such sensitized emission was first proposed with a phosphor as sensitizer (*33*), known as phosphor-sensitized fluorescence (PSF). However, the FRET process in PSF is intrinsically limited by the relatively low *k*_r_ (~10^5^ to 10^6^ s^−1^) of phosphors as the Förster interaction is proportional to the *k*_r_ of the sensitizer (*35*). Later, an alternative strategy using TADF materials as sensitizers was separately proposed by our group and Adachi’s group, known as TADF-sensitized fluorescence (TSF) or hyperfluorescence (*4*, *34*). TADF sensitizers usually have large *k*_r_s (~10^6^ to 10^7^ s^−1^) and, thus, high FRET rates (*k*_FRET_s) of more than 10^7^ s^−1^, which promises the fast exciton consumption. It is commonly believed that the overall radiative dynamic process of TSF is limited by the inefficient reverse intersystem crossing rate (*k*_RISC_) (*36*), and, thus, plenty of efforts have been devoted to developing TADF sensitizers that achieve high *k*_RISC_s of ~10^6^ to 10^7^ s^−1^ (*28*, *37*). Despite all those efforts, previously reported TSF devices still suffer from long-delay tails under electrical excitation, which induce significant efficiency roll-off, and no explanation has been proposed (*38*).

Theoretically, for a TADF sensitizer, a fast *k*_RISC_ is always accompanied by an even faster intersystem crossing rate (*k*_ISC_), which can achieve ~10^7^ to 10^8^ s^−1^. Therefore, although having been ignored in previous studies, the intersystem crossing process is a strong competitor of the FRET process. In this scenario, not all singlet excitons formed on the sensitizer will be transferred to the dopant in a one-way process. Instead, plenty of TADF excitons will repeat the singlet-triplet spin-flip cycles until they are finally transferred to the dopant as illustrated in fig. S1. Hence, the spin-flip cycle will result in long-delay tails, particularly under electroluminescence excitation endowing 75% of triplet and only 25% of singlet formation. The situation becomes more difficult when the mutual exclusion of both large *k*_RISC_ and *k*_r_ in TADF material is considered.

To circumvent this issue, we propose an advanced TPSF strategy that contains a ternary system—a TADF host, a phosphor assistant, and a fluorescent dopant. As illustrated in Fig. 1B, besides the TADF-sensitized process, an additional Dexter energy transfer (DET) from the triplet of the TADF host to that of the phosphor can be anticipated in TPSF, and the energy will then be transferred to the dopant. It offers a one-way exciton consumption of the TADF host without competing cycles. Given the distance-dependent characteristics of both the DET and FRET process, manipulation of exciton dynamics can be achievable by optimizing the concentrations of both the phosphor and dopant in TPSF. In this way, the singlet-triplet spin-flip cycles of the TADF host can be broken to accelerate the exciton emissive dynamics compared with the sole TSF strategy.

Besides the fast radiative dynamics, high exciton utilization efficiency is also required for ideal device stability as aforementioned. The plausible exciton loss pathways in the TPSF system are mainly through the triplet states of fluorescent dopants, formed by either direct charge recombination on dopants or the DET from the host/sensitizer to the dopants. It has revealed that sterically encapsulated dopants can not only reduce charge trapping but also alleviate Dexter interactions, blocking exciton loss in the sensitization system (*33*, *39*). Therefore, by using proper dopants, devices based on the TPSF system are viable to realize high exciton utilization efficiency as well as a fast consumption rate in a nanosecond regime.

What needs to be further noticed is that the greatly accelerated radiative decay process can be realized via multiple sensitizations in TPSF; however, there is a great chance that the formation rate of excitons will eventually exceed their consumption rate under ultrahigh luminance. It is indicated that exciton saturation of dopants and, thus, excessive excitons of host can be anticipated. Considering the short intermolecular distance among host molecules, host-involved annihilations will emerge that impede the ultimate elimination of the efficiency roll-off in OLED—yet it has been underestimated in previous works. One plausible solution is to adopt a TADF host with an anti-ACQ character, which has been proved to intrinsically suppress the relevant annihilations (*40*).

**Fig. 2.**
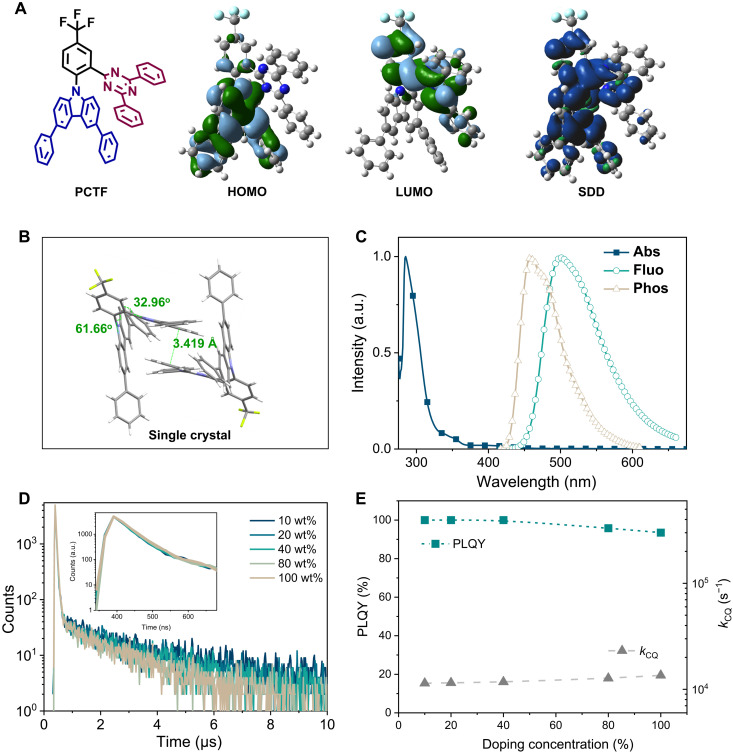
Molecular structure and photophysical characterizations. (**A**) Molecular structure, HOMO/LUMO distribution, and *T*_1_ SDD of PCTF. (**B**) Molecular packing of PCTF in a single crystal characterized by x-ray diffraction. (**C**) Absorption, fluorescence, and phosphorescence spectra of PCTF in toluene solution (10^−5^ M). (**D**) Photoluminescence transient decay curves of PCTF–9-(3-(9*H*-carbazol-9-yl)phenyl)-9*H*-3,9′-bicarbazole (mCPCz)–doped films with varying concentrations (inset figure of partially enlarged view). (**E**) Photoluminescence quantum yield (PLQY) and quenching rate (*k*_CQ_) of PCTF-mCPCz–doped films with varying concentrations.

To meet the above requirements, a TADF material, 9-(2-(4,6-diphenyl-1,3,5-triazin-2-yl)-4-(trifluoromethyl) phenyl)-3,6-diphenyl-9H-carbazole (PCTF), was developed as the host, the structure of which is provided in Fig. 2A. This compound uses 3,6-diphenyl-carbazole as the donor and 2,4-diphenyl-1,3,5-triazine as the acceptor, appended on a core phenyl ring in an ortho-position manner with a trifluoromethyl unit at the para-position of the donor to tune optoelectronic properties. The geometric and electronic properties of this compound were performed on the basis of density functional theory (DFT) at the B3LYP/6-31G(d) level. A highly twisted structure was observed owing to the sterical hindrance. The distribution of the highest occupied molecular orbital (HOMO) and the lowest unoccupied molecular orbital (LUMO) was spatially separated, with the former mainly on the 3,6-diphenyl-carbazole unit and the latter across the triazine unit and core phenyl ring, as illustrated in Fig. 2A. The spatially separated HOMO/LUMO distribution facilitated a small singlet-triplet energy gap (Δ*E*_ST_), which was predicted by the time-dependent DFT to be 0.06 eV, with the lowest singlet (*S*_1_) energy of 2.62 eV and the lowest triplet (*T*_1_) energy of 2.56 eV, respectively. Besides, some HOMO/LUMO overlap on the core phenyl ring was observed, which explained a moderate oscillator transition strength of 0.0149. Furthermore, DFT calculation for the lowest excited triplet state of the PCTF molecule was also performed and revealed that the spin density distribution (SDD) resided on the D-A molecular structure, except for the two phenyl rings of the 2,4-diphenyl-1,3,5-triazine unit in Fig. 2A.

The single crystal structure of PCTF is depicted in Fig. 2B, illustrating dihedral angles of 32.96°/61.66° between the triazine/carbazole plane and core phenyl, respectively. In addition, a weak π-π stacking with a distance of 3.419 Å between phenyl planes of 2,4-diphenyl-1,3,5-triazine units in adjacent molecules was observed. As aforementioned, no SDD of *T*_1_ resided on those phenyl rings. Therefore, those phenyl rings will act as inert units to enlarge intermolecular distances. Previous work has revealed that the concentration quenching of TADF molecules is mainly dominated by the electron-exchange interactions of triplet excitons, known as the Dexter model (*40*). Regarding the short-range interaction nature of DET, it has been proved that even a small modulation of molecular geometric structure will markedly affect the concentration-quenching behaviors. Therefore, it is reasonable that the highly twisted structure and inert protection of PCTF could effectively suppress the intermolecular Dexter interactions for anti-ACQ property. Then, the energy levels of PCTF were measured by cyclic voltammetry as illustrated in fig. S2B, revealing a HOMO level of −5.68 eV and a LUMO level of −2.81 eV, respectively.

Photophysical properties of PCTF were measured in toluene with a concentration of 10^−5^ M as summarized in Table 1. As depicted in Fig. 2C, ultraviolet-visible (UV-vis) absorption illustrated a sharp peak at 285 nm, which should arise from the π-π^*^ transition of the carbazole unit. In addition, a weak wide absorption peaking around 400 nm was also observed, assigned to the charge transfer transition of PCTF. This charge transfer nature leads to a featureless wide emission spectrum peaking at 490 nm, of which the onset defines the singlet energy of 2.75 eV. Phosphorescent spectrum with a less-defined structure was recorded at 77 K, indicating the mixed charge transfer and localized excited character. A *T*_1_ energy of 2.71 eV was then obtained from the emission peak, which resulted in a small Δ*E*_ST_ of 0.04 eV, favoring the efficient RISC process.

**Table 1.** Photophysical characteristics of PCTF.

	PL_max_^*^ (nm)	**HOMO/LUMO (eV)**	***S*_1_/*T*_1_ (eV)**	Ф_p_^†^/Ф_d_^*^	τ_p_^*^ (ns)	τ_d_^*^ (μs)	***k*_r_ (10^6^ s^−1^)**	***k*_ISC_ (10^7^ s^−1^)**	***k*_RISC_ (10^6^ s^−1^)**
PCTF	499	−5.68/−2.81	2.75/2.71	0.33/0.67	45.6	3.65	7.24	1.47	0.83

*Measured in degassed toluene solution of 10^−5^ M at 298 K.

^†^Measured in toluene solution of 10^−5^ M under ambient condition at 298 K.

The concentration-quenching character of PCTF was further evaluated in doped films using a wide–energy gap host, 9-(3-(9*H*-carbazol-9-yl)phenyl)-9*H*-3,9′-bicarbazole (mCPCz), with varied dopant concentrations from 10 to 100 weight % (wt%) (pristine film). In addition, the photoluminescence transient decay curves of those films were measured and provided in Fig. 2D. The lifetimes of the prompt parts remained unchanged at 44 ns for all decay curves, as illustrated in the inset figure with a narrower time range, verifying the negligible effect that singlet took on the concentration quenching. Only slightly reduced lifetimes were recorded for the delayed components of all those decay curves, which indicated the effective blocking of the nonradiative quenching pathways for the triplet excitons. The alleviated exciton annihilation character accounts for the high photoluminescence quantum efficiencies (PLQYs) of more than 90% for all films, as depicted in Fig. 2E, validating the anti-ACQ character of PCTF.

We further examined the rate constant of concentration quenching (*k*_CQ_) as a function of dopant concentration in the doped films. The *k*_CQ_ was obtained on the basis of the equation (*40*)

$$k_{\text{CQ}}=\frac{1}{2}(k_{\text{PF}}+k_{\text{DF}}-2k_{\text{RISC}}-\sqrt{{\left(k_{\text{PF}}-k_{\text{DF}}\right)}^{2}-4k_{\text{ISC}}k_{\text{RISC}}})$$

where *k*_PF_ and *k*_DF_ refer to the prompt fluorescence rate and the delayed fluorescence rate, respectively. The *k*_CQ_-dopant concentration character is provided in Fig. 2E, showing a monotonic increment of *k*_CQ_ with increasing dopant concentration. In addition, even for the pristine film, a relatively low *k*_CQ_ of only 1.35 × 10^4^ s^−1^ was observed. Meanwhile, the *k*_RISC_ and *k*_r_ of PCTF in the pristine film were as high as 8.30 × 10^5^ and 7.24 × 10^6^ s^−1^, respectively. All the above results suggest that PCTF has a high PLQY, a fast radiative decay, and a significant anti-ACQ character, promising it as an ideal TADF host for the TPSF system.

### Energy transfer analysis of the doped films

**Fig. 3.**
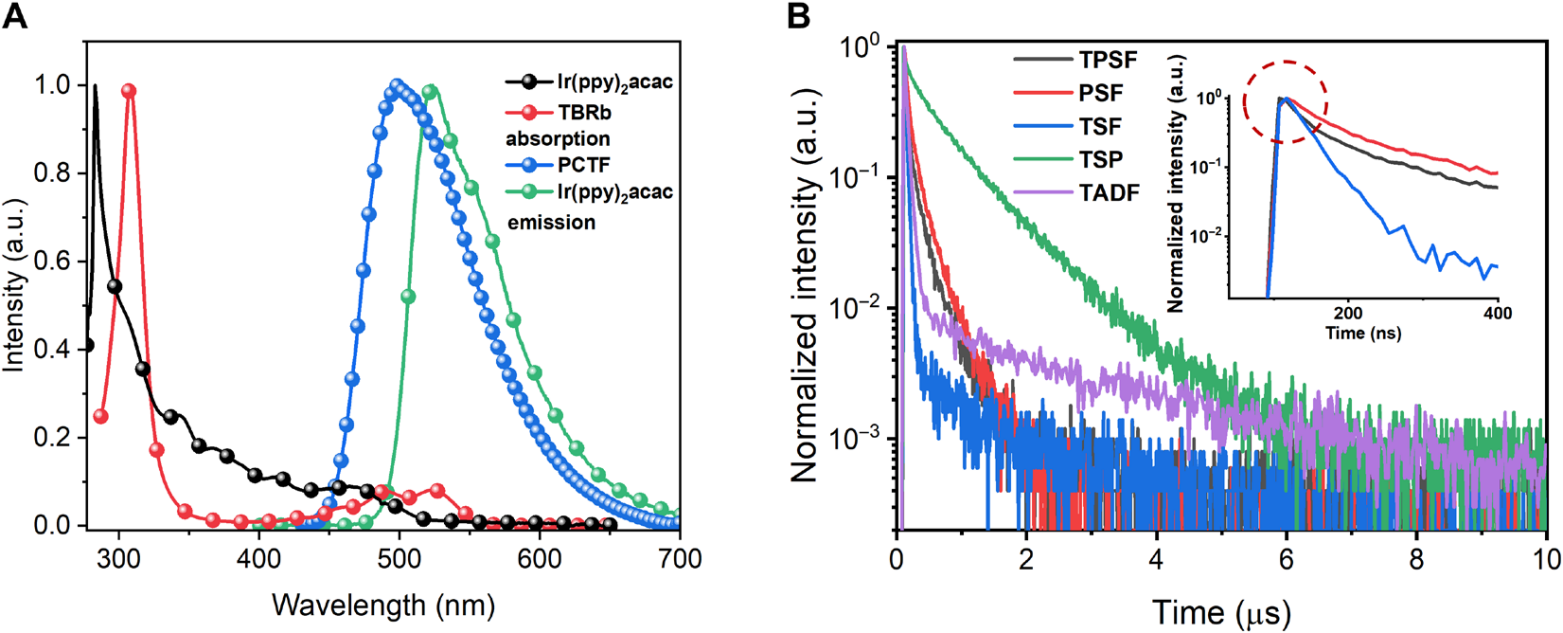
Energy transfer analysis. (**A**) Absorption spectra of Ir(ppy)_2_acac/TBRb and PL spectra of PCTF and Ir(ppy)_2_acac in toluene solution at room temperature. (**B**) PL transient decay curves of all compared systems measured at the dominant emission peak (inset figure of partially enlarged view). TPSF [PCTF: 20 wt% Ir(ppy)_2_acac: 1 wt% TBRb], TSP [PCTF: 20 wt% Ir(ppy)_2_acac], TSF (PCTF: 1 wt% TBRb), PSF [CPB: 20 wt% Ir(ppy)_2_acac: 1 wt% TBRb], and TADF (PCTF pristine film).

To maximize the performance of TPSF, the phosphor assistant and fluorescent dopant were also elaborately chosen as bis[2-(2-pyridinyl-*N*)phenyl-C](acetylacetonato)iridium(III) [Ir(ppy)_2_(acac)] (*41*) and 2,8-di-*tert*-butyl-5,11-bis(4-*tert*-butylphenyl)-6,12-diphenyltetracene (TBRb) (*42*), respectively. Figure S4 illustrates the photophysical properties of both compounds measured in degassed toluene with a concentration of 10^−5^ M. Ir(ppy)_2_acac exhibited a moderate exciton lifetime of 1044 ns, with a high PLQY of 93%, accounting for a moderate *k*_r_ of 0.9 × 10^6^ s^−1^. Meanwhile, TBRb afforded a short radiative decay lifetime of ~15 ns and a high PLQY of 95%, leading to a large *k*_r_ of 6.3 × 10^7^ s^−1^. In addition, the HOMO/LUMO and *T*_1_ SDD of TBRb were perfectly confined on rubrene by the *tert*-butyl units as illustrated in fig. S4D, which has been proved to effectively inhibit the relevant DET (*43*) and eliminate the exciton loss pathway. Figure 3A shows the UV-vis absorption spectrum of TBRb and Ir(ppy)_2_acac as well as the emission spectra of PCTF and Ir(ppy)_2_acac in toluene solution at 298 K. Apparently, multiple spectral overlaps can be observed, which promises several efficient FRET processes. Critical FRET radii (*R*_0_s) were calculated as 4.8 nm for PCTF → TBRb, 4.6 nm for Ir(ppy)_2_acac → TBRb, and 3.0 nm for PCTF → Ir(ppy)_2_acac, which favor efficient sensitizations.

To elucidate the sensitizing processes, photoluminescence decay curves of TSF films (PCTF: TBRb) and PSF films [CBP: 20 wt% Ir(ppy)_2_acac: TBRb] were measured, where CBP is 4,4′-bis(*N*-carbazolyl)-1,1′-biphenyl, a wide–energy gap host without a TADF character. The concentrations of TBRb were 0, 0.5, 1.0, and 1.5 wt%. Figure S3 illustrates the gradually shortened exciton lifetimes of sensitizers, with the increasing concentration of TBRb in both TSF and PSF films. Those phenomena have been regarded as the reflection of the energy transfer—sensitization—from sensitizer to dopant (*33*). Regarding the specific energy transfer rates (*k*_FRET_s), 1.3 × 10^7^ and 3.2 × 10^6^ s^−1^ were recorded for 1 wt% TBRb-doped TSF and PSF films, respectively, as shown in table S2. The larger *k*_FRET_s in TSF films ascribed to the higher *k*_r_ of PCTF compared with Ir(ppy)_2_acac. Although endowing a higher *k*_FRET_, a clear delayed tail still exists in the decay curve of the TSF film observed at the emission peak of TBRb, which is different with the PSF film as depicted in Fig. 3B. We attributed this to the high *k*_ISC_ of PCTF (1.47 × 10^7^ s^−1^), which is comparable with *k*_FRET_. Specifically, the ISC process will strongly compete with the FRET process to induce singlet-triplet spin-flip recycles as aforementioned and finally leads to the long-delay tail. This situation is even worse under electrical excitation, because the ratio of directly formed triplet is as high as 75%. In contrast, the sensitizing process in the PSF film is a one-way process and, thus, afforded a single-exponent decay without delay.

The photoluminescence decay curve of TPSF was also measured and depicted in Fig. 3B. It exhibited a nanosecond-level lifetime that was shorter than that of PSF but longer than the prompt one of the TSF system, evidencing multiple energy transfers. Theoretically, the photoluminescence decay curves should reflect the overall dynamics of not only singlet but also triplet exciton, as both of them were involved in the emissive processes. The shortened lifetime of the TPSF decay curve therefore verified the accelerated consumption of both triplet and singlet excitons. No clear long-delay tail was observed in the TPSF film, suggesting that the additional Dexter interaction significantly broke the singlet-triplet spin-flip recycles of the TADF host and, thus, accelerated the exciton consumption as anticipated.

### OLED performances

**Fig. 4.**
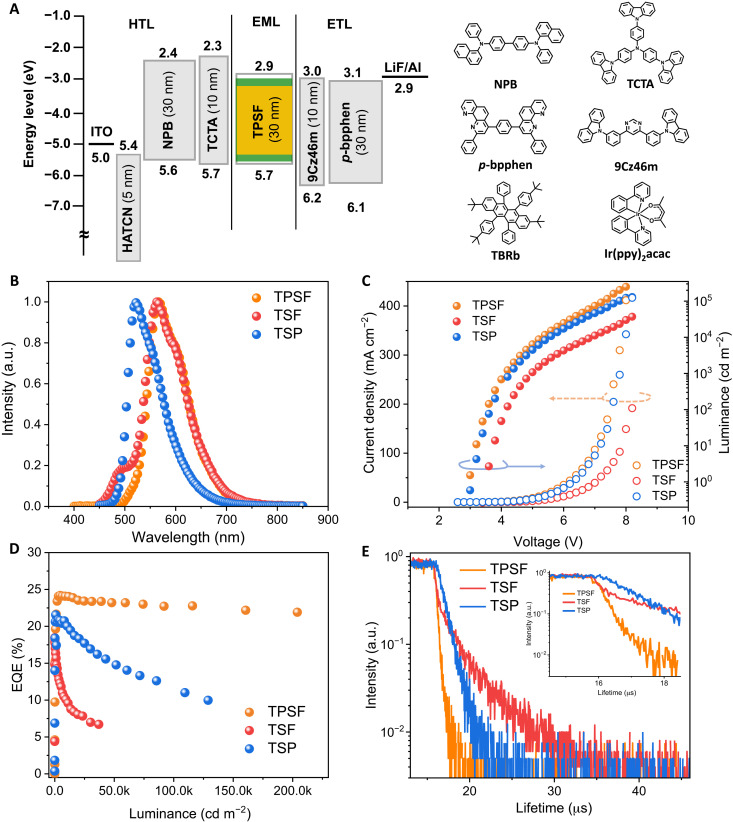
OLED device performance. (**A**) The energy diagrams and molecular structures of the device. (**B**) EL spectra of all devices measured at 10,000 cd m^−2^. (**C**) Current density-voltage-luminance (*J*-*V*-*L*) curves of all devices. (**D**) EQE-luminance curves of devices. (**E**) EL transient decay curves measured at the dominant emission peak with a driving voltage of 6 V (inset figure of partially enlarged view).

To testify the rationality of this TPSF system, OLEDs featuring structures of indium tin oxide (ITO), dipyrazino[2,3-f:2′,3′-h]quinoxaline-2,3,6,7,10,11-hexacarbonitrile (5 nm), *N*,*N*′-di(1-naphthyl)-*N*,*N*′-diphenyl-(1,1′-biphenyl)-4,4′-diamine (30 nm), tris(4-carbazoyl-9-ylphenyl)amine (10 nm), the emitting layer (EML) (24 nm), 4,6-bis(3-(9*H*-carbazol-9-yl)phenyl)pyrimidine (10 nm), 1,4-bis(2-phenyl-1,10-phenanthrolin-4-yl)benzene (30 nm), LiF (0.7 nm), and Al (150 nm) were prepared, where EML was composed of PCTF: 20 wt% Ir(ppy)_2_acac: 1 wt% TBRb (TPSF). Contrast devices using PCTF: 20 wt% Ir(ppy)_2_acac [TADF-sensitized phosphorescence (TSP)] and PCTF: 1 wt% TBRb (TSF) as EML were also fabricated for comparison. Table 2 summarizes the relevant device performance. The diagram of the device energy levels and the adopted molecule structures are illustrated in Fig. 4A.

**Table 2.** Performance summary of all types of OLED devices. PE, power efficiency; CIE, Commission Internationale de l’Eclairage color coordinate.

**Device**	**EQE (%)**				***L*_90%_ (cd m^−2^)**	**PE_max_ (lm W^−1^)**	**CIE**	LT85^*^ (1000 cd m^−2^) (hours)
	**Max**	**10,000 cd m^−2^**	**50,000 cd m^−2^**	**100,000 cd m^−2^**				
**TPSF**	24.2	24.0	23.2	22.7	190,500	50.2	(0.49,0.50)	13,215
**TSP**	21.6	20.1	14.8	11.1	13,280	60.5	(0.34,0.61)	674
**TSF**	18.4	9.7	-	-	720	44.2	(0.47,0.49)	140
**PSF**	21.9	13.4	-	-	1,600	64.3	(0.48,0.51)	2,249

*Converted by LT85 (1000 cd m^−2^) = LT85 (10,000 cd m^−2^) **×** ${\left(\frac{10,000\mathrm{cd}m^{-2}}{1000\mathrm{cd}m^{-2}}\right)}^{n}$, with *n* = 1.75.

Electroluminescence (EL) spectra of all devices at 10,000 cd m^−2^ are depicted in Fig. 4B. Note that clear emission from PCTF at 500 nm was observed besides the dominant emission peaking at 560 nm from TBRb in the TSF device (fig. S5A), which was enhanced with increasing voltages and, thus, suggested the incomplete energy transfer. In contrast, the TPSF device that showed main orange emission peaking at 560 nm solely comes from TBRb, which remained the same under different luminances as provided in fig. S5B. Those results indicated the great role of phosphor assistant in improving the energy transfer process. Figure 4C provides the current density–voltage–luminance (*J*-*V*-*L*) characteristics of those devices. The relatively lower emission intensity of the TSF device suggested a low exciton utilization efficiency, which was evidenced by the relatively low EQE_max_ of only 18.4% as illustrated in Fig. 4D. As a comparison, a high EQE_max_ of 24.2% was obtained for the TPSF device. As aforementioned, in the sensitized system, the exciton loss pathway is mainly attributed to the DET process from the host/sensitizer to the fluorescent dopant. The relatively higher exciton utilization efficiency in the TPSF device should arise from the more efficient energy transfer compared with the TSF device. Besides, a high ratio of horizontal orientation of emitting dipole was also recorded for TBRb as shown in fig. S4C, which, thus, explains the relatively higher light outcoupling efficiency than the predicted one of 20%. Besides, the EQE_max_ of the TPSF device was even higher than the TSP device with an EQE_max_ of 21.6%. In addition to the high efficiency for the TPSF device, it was truly remarkable that an extremely low efficiency roll-off was observed, presenting an ultrahigh *L*_90%_ of >190,500 cd m^−2^ with a maximum luminance of 249,300 cd m^−2^. To the best of our knowledge, this is the highest *L*_90%_ ever reported in bottom-emitting OLEDs, as illustrated in Fig. 1A. As a comparison, *L*_90%_ of merely 13,280 and 720 cd m^−2^ were recorded for TSP and TSF, respectively, both exhibiting significant efficiency roll-off. Those results validate the effectiveness and advantages of TPSF in eliminating the efficiency roll-off with increasing luminance.

To unravel the origination of the different roll-off behaviors from the point of exciton dynamics under electrical excitation, EL transient decay curves of those devices are provided in Fig. 4E, which were measured at the dominant emission peaks. In terms of the TSF device, after a fast prompt part with a fitted lifetime of 370 ns and a ratio of only 22%, a long-delay tail with a lifetime of 3050 ns and a rather high ratio of 78% was observed. The lifetime of the delayed component was even longer than that of the TSP device and, thus, explained the worst efficiency roll-off of the TSF device. The delayed components should arise from the competitive ISC process as aforementioned, which leads to singlet-triplet recycles of the TADF host. For the TSF device, the delayed component of the EL decay curve was even more significant than the photoluminescence excitation, confirming our earlier speculation. By increasing the dopant concentration in TSF devices, the FRET can be enhanced, but the long-delay tails still exist as depicted in fig. S6H, which evidenced the great influence of the ISC process. Rapid exciton consumption with a fitted lifetime of only 203 ns without clear delayed component was observed for the TPSF device. Theoretically, the exciton lifetime depends on both radiative and nonradiative decay processes. Given the high EQE_max_ of the TPSF device, the nonradiative decay can be reasonably ruled out. The reason should be attributed to the phosphor assistant, which greatly reduced the numbers of singlet-triplet recycles on the TADF host and induced an additional energy transfer process to dopant. As a result, both the rapid exciton radiative dynamics and the high exciton utilization efficiency should account for the ultrahigh *L*_90%_ of the TPSF device.

To testify the critical role of the TADF host, we also developed PSF contrast devices using CBP as the non-TADF host, and the concentration of the phosphor assistant was optimized as illustrated in fig. S7. The EL spectra of those devices showed dominant emission from TBRb, but phosphor emission remained, which gradually reduced with increasing phosphor concentration, indicating the incomplete energy transfers. The *J*-*V*-*L* curves of those devices exhibited clear dependence on phosphor concentrations, suggesting that the main recombination was through charge trapping on phosphor. Both the EQE_max_ and the efficiency roll-off of those devices improved by increasing the phosphor concentrations. In the PSF device using 20 wt% Ir(ppy)_2_acac and 1 wt% TBRb, an EQE_max_ of 21.9% and an *L*_90%_ of only 1600 cd m^−2^ were recorded for comparison with the TPSF device. The EL decay curves of those devices showed relatively long exciton lifetimes, which should be attributed to the relatively low *k*_FRET_ from phosphor to dopant. Besides, with increased phosphor concentrations, a clear trend of decreased exciton lifetimes was observed that mainly arose from the shortened phosphor-dopant molecular distance and, thus, enhanced FRET process. In summary, the overall performances of PSF devices were much inferior to those of TPSF devices, as both the relatively slow exciton consumption and direct charge trapping on phosphor exacerbated the efficiency roll-off, which was comprehensively studied by Lee *et al.* (*44*). What is important but has always been neglected is that excitons directly recombine on CBP, which will inevitably happen under extremely high luminance, which may induce host-exciton–involved annihilations for further efficiency loss. By using the TADF host, not only quick exciton consumption due to the efficient FRET but also the wide recombination zone direct on host with narrow energy gap can be anticipated, both helping to suppress exciton annihilations. In addition, the TADF host adopted in this work exhibited a strong anti-ACQ character, which should also contribute to the suppression of exciton annihilations.

To further testify the universality of this TPSF strategy, previously reported TADF materials, 10-(4-(4,6-diphenyl-1,3,5-triazin-2-yl)phenyl)-9,9-dimethyl-9,10-dihydroacridine (DMAC-TRZ) and bis(4-(9,9-dimethylacridin-10(9H)-yl)phenyl)methanone (DMAC-BP), were also chosen as hosts to construct sensitized devices. The specific device performances are provided in fig. S9. Notably, large EQE_max_s of 24.8 and 20.2% as well as high *L*_90%_s of 129,000 and 101,000 cd m^−2^ were obtained for the DMAC-TRZ– and DMAC-BP–based TPSF devices, respectively. Both TPSF devices significantly outperform others with only phosphor or fluorophor as emitters, of which the EL decay curves also revealed the fast exciton consumption processes, evidencing the reliability of the TPSF strategy.

**Fig. 5.**
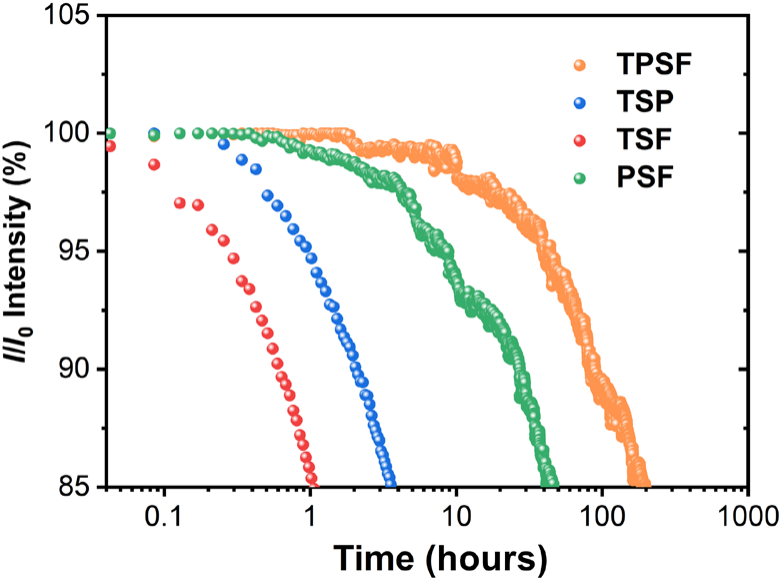
Device lifetimes of all devices with an initial luminance of 10,000 cd m^−2^.

Next, we assessed the operational stability of the fabricated devices. Figure 5 displays the luminance of those OLEDs as a function of operational time under constant current density with an initial luminance (*L*_0_) of 10,000 cd m^−2^. A remarkable enhancement of operational stability in the TPSF OLED was observed, resulting in an *LT85* (the lifetime of luminance decays to 85% of its *L*_0_) of 235 hours. In contrast, a rapid decrease of luminance with increasing operation time was observed for TSP and TSF devices, of which the *LT85*s were only 12 and 2.5 hours, respectively. Besides, the PSF device showed inferior stability with an *LT85* of 40 hours, only about one-sixth of the TPSF life span. In analogy to efficiency roll-off, the degradation of OLEDs has also been convincingly attributed to annihilation between excited states, which finally brings about energetically hot excited states to dissociate molecule bonds. The quick radiative assumption of excitons in the TPSF device should also contribute to device stability in addition to alleviated efficiency roll-off. The converted *LT85* of the TPSF device at an initial luminance of 1000 cd m^−2^ can be extrapolated using the following equation: *LT85* (1000 cd m^−2^) = *LT85*(10,000 cd m^−2^) × ${\left(\frac{10,000\;\text{cd}\;m^{-2}}{1000\;\text{cd}\;m^{-2}}\right)}^{n}$, with a degradation acceleration factor (*n*) of 1.75, resulting in 13,215 hours. Those results validate the reliability of the TPSF strategy in improving both high efficiency and device stability, which is highly desired for practical applications where high luminance is required.

## DISCUSSION

The efficiency roll-off at high luminance induced by exciton annihilations has hindered the wide application of OLEDs for decades. Combining high exciton utilization efficiency and short exciton residence lifetime may tackle this issue, which, however, cannot be satisfied by the present emitters solely. The TSF emission provides a rational solution, but the competitive ISC process of the TADF sensitizer always leads to singlet-triplet spin-flip cycles and, thus, slows the exciton consumption in such strategy.

In this work, we proposed a TPSF strategy, incorporating an assistant phosphor into the TSF system, to elaborately break the above cycles for an efficient and fast radiative decay via multiple sensitizations. In addition, benefiting from the anti-ACQ character of the TADF-sensitizing host, a roll-off–free OLED was fabricated, endowing an ultrahigh *L*_90%_ of more than 190,500 cd m^−2^, a high EQE_max_ of 24.2%, and a remarkable *LT85* of 13,215 hours. Given that the long-lived EL exciton still exhibits a lifetime of hundred nanoseconds in the current TPSF device, there is still significant room for further improvement of exciton dynamics. One plausible solution can be the use of multi-resonance (MR) materials as dopant, which has an ultrafast *k*_r_ of more than 10^8^ s^−1^ such that the upper limit of device luminance and stability should be further improved by virtue of significantly accelerated exciton consumption, for expanded application fields. It can be anticipated that devices with exciton lifetimes in tens of nanoseconds and 100% exciton utilization may be finally realized on the basis of further material development and device structure optimization, leading to further reduced roll-off and prolonged device lifetime.

Recently, Kim *et al.* proposed an exciplex-hosted PSF OLED device, in which exciplex and phosphor realized exciton conversion and energy transfer to a fluorescent dopant (*6*). However, despite the inefficient TADF characteristics of the exciplex-forming host, a clear efficiency roll-off was still observed under an ultrahigh luminance of more than 10,000 cd m^−2^. Besides, a four-component EML would significantly increase the complexity of device fabrication and harm its reproducibility. Our strategy here not only reveals the multiple sensitization mechanism in detail but also shows superiority in eliminating efficiency roll-off by highlighting the critical role of an anti-ACQ TADF host and simplifying the device fabrication, thus paving a solid way for the practical applications of OLEDs in the fields where ultrahigh luminance is required. In particular, as a game-changing technology, organic solid-state lasers have attracted a lot of attention recently. Despite the successful amplified spontaneous emission by optical pumping, electrically driven organic laser devices continue to suffer from the long-lived triplet excitons that lead to optical losses. Scavenging triple excitons by a triplet quencher is the most used solution, which, however, is counterproductive for the electrically pumped organic laser, as a four times higher threshold current density is required compared with the ideal case that can realize 100% exciton utilization efficiency (*45*). The key to resolve this issue is to constructively use triplet excitons in a rapid manner. The TPSF strategy proposed here may open a new avenue toward electrically pumped organic laser devices regarding its ability to harness triplet excitons in a nanosecond or an even shorter regime, which may greatly reduce the threshold current density while improving device stability.

## MATERIALS AND METHODS

### General

All commercial reagents were used without further purification. ^1^H nuclear magnetic resonance spectra were measured with a Bruker 600-MHz spectrometer in which tetramethylsilane was used as the internal standard. Mass spectra were recorded using matrix-assisted laser desorption ionization time-of-flight mass spectrometry (Shimadzu, Japan). Electrochemical measurements were performed with a CHI600E electrochemical workstation with a scan rate of 100 mV s^−1^ in which Pt was used as the working electrode, platinum wire was used as the auxiliary electrode, and an Ag/AgCl system was used as the reference electrode, while ferrocene/ferrocenium was used as the standard. Anhydrous dichloromethane and *N*,*N*-dimethylformamide solution were applied for oxidation and reduction potential, respectively. The LUMO energy level was determined from the cathodic reduction potentials, while the HOMO energy level was determined from the oxidation potentials. All doping concentrations in this paper are mass fractions.

### Instrument information

UV-vis absorption spectra were measured using an Agilent 8453 (Palo Alto, California, USA) spectrophotometer. PLQYs and device efficiencies were measured in air atmosphere by an absolute PL quantum yield measurement system (C9920-02; Hamamatsu Photonics, Shizuoka, Japan), with an excitation wavelength of 360 nm. The fluorescent and phosphorescent spectra were characterized in 10^−5^ M toluene solution using an Edinburgh Instruments LP920-KS fluorescence spectrophotometer. The photoluminescence transient decay curves of the films and device electroluminescence transient decay curves were recorded, which used a transient spectrometer (Edinburgh FLS1000, Edinburgh, Britain). Device lifetimes were measured using an OLED aging lifetime tester (ZJZCL-1, Shanghai University).

### Device fabrication

All ITO glass substrates were carefully precleaned, and all organic materials were purified by vacuum sublimation before device fabrication. The devices were prepared in vacuum at a pressure of 2 × 10^−6^ torr. All organic materials were thermally evaporated at a rate of <1.0 Å s^−1^ After cooling for 30 min, device encapsulation was carried out in a glove box following performance characterizations and lifetime measurements.

## Supplementary Materials

### This PDF file includes:

Synthesis pathway Calculation equation of FRET radius Figs. S1 to S9 Tables S1 to S3
