# Supplementary material for: Highly efficient and nearly roll-off–free electrofluorescent devices via multiple sensitizations
Source: Sci Adv. 2022 Jul 27;8(30):eabp9203. doi: 10.1126/sciadv.abp9203 (PMC9328673; doi:10.1126/sciadv.abp9203)
Supplement: Supplementary file 1 — Synthesis pathway Calculation equation of FRET radius Figs. S1 to S9 Tables S1 to S3 [file sciadv.abp9203_sm.pdf]

Science Advances
AAAS

Supplementary Materials for

Highly efficient and nearly roll-off–free electrofluorescent devices via multiple sensitizations

Chen Yin *et al.*

Corresponding author: Email: Dongdong Zhang, ddzhang@mail.tsinghua.edu.cn

Sci. Adv. **8**, eabp9203 (2022)
DOI: 10.1126/sciadv.abp9203

This PDF file includes:

Synthesis pathway
Calculation equation of FRET radius
Figs. S1 to S9
Tables S1 to S3

Synthesis pathway

Synthesis of 2-(2-fluoro-5-(trifluoromethyl)phenyl)-4,6-diphenyl-1,3,5-triazine (CF3-TRZ)

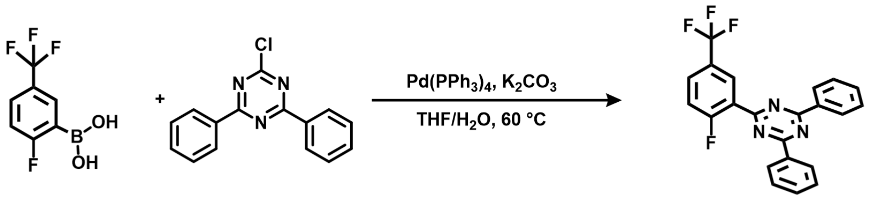

A mixture of (2-fluoro-5-(trifluoromethyl)phenyl)boronic acid (2.07g, 10 mmol), 2-chloro-4,6-diphenyl-1,3,5-triazine (2.68 g, 10 mmol), Tetrakis(triphenylphosphine)palladium (0.58 g, 0.5 mmol) and potassium carbonate (5.53 g, 40 mmol) in 60 ml THF/H_2O (volume ratio 2:1) was added into a three-necked bottle and stirred at 60 °C overnight under nitrogen atmosphere. Then the reaction was cooled to room temperature and poured into water, followed with extraction by dichloromethane. Next, the combined organic layer was dried with anhydrous Na_2SO_4, and the solvent was removed under reduced pressure. The residue was purified by column chromatograph using CH_2Cl_2/petroleum ether (1:3) as the solvent to get 2-(2-fluoro-5-(trifluoromethyl)phenyl)-4,6-diphenyl-1,3,5-triazine (CF3-TRZ) as a white solid (3.2 g, 80%).

Synthesis of 9-(2-(4,6-diphenyl-1,3,5-triazin-2-yl)-4-(trifluoromethyl)phenyl)-3,6-diphenyl-9H-carbazole (PCTF)

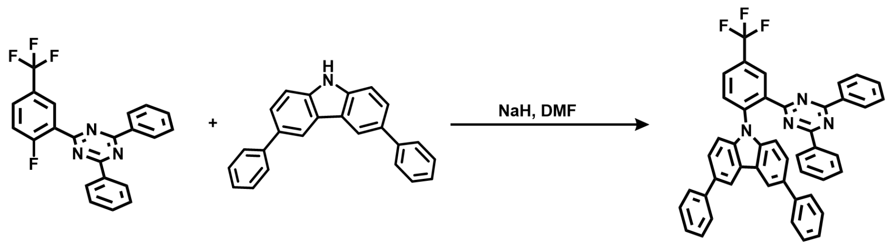

NaH (60% in mineral oil) (0.4 g, 10 mmol) was dispersed in 30 ml Dimethylformamide (DMF) in an ice bath, and then 10 ml DMF solution of 3,6-diphenyl-9H-carbazole (1.92 g, 6 mmol) was added into the system dropwise under stirring. The mixture was stirred for 30 minutes at room temperature. Next, 10 ml DMF solution of CF3-TRZ (1.98 g, 5 mmol) was added into the system dropwise and heated to 60 °C for 1 hour. Then the mixture was cooled to room temperature and then poured into water. Precipitate was collected and then dried under vacuum. Then the crude product was further purified by column chromatography with CH_2Cl_2/petroleum ether (1:2) as the solvent to get 9-(2-(4,6-diphenyl-1,3,5-triazin-2-yl)-4-(trifluoromethyl)phenyl)-3,6-diphenyl-9H-carbazole (PCTF) as a bright-yellow solid (3.1 g, 90%). ^{1}H NMR (600 MHz, $CDCl_3$-d + TMS, 298 K): δ (ppm) = 8.80 (1H, s), 8.25 (2H, s), 8.10 (1H, d), 8.03 (4H, d), 7.91 (1H, d), 7.66 (4H, d), 7.58 (2H, d), 7.45 (6H, m), 7.33 (2H, t), 7.28 (6H, m).

Calculation of FRET radius

$$R_0^6 = \Phi_D . \kappa^2 \left(\frac{9000. ln10}{128. \pi^5 . N_A . n^4}\right) . \int_0^\infty F_D(\lambda). \varepsilon_A(\lambda). \lambda^4 d\lambda$$

The specific FET radii (R_0s) were calculated by the above equation, in which Φ_D is the photoluminescence quantum efficiency of donor; κ^2 is the configurational factor between donor and acceptor (assumed to be 2/3 with regard of random distribution); N_A is the Avogadro's constant; n is the refractive index of the medium and $F_D(\lambda)$ is the emission spectra of donor with normalized area; $\varepsilon_A(\lambda)$ is the molar absorption coefficient (mol m^{-4}).

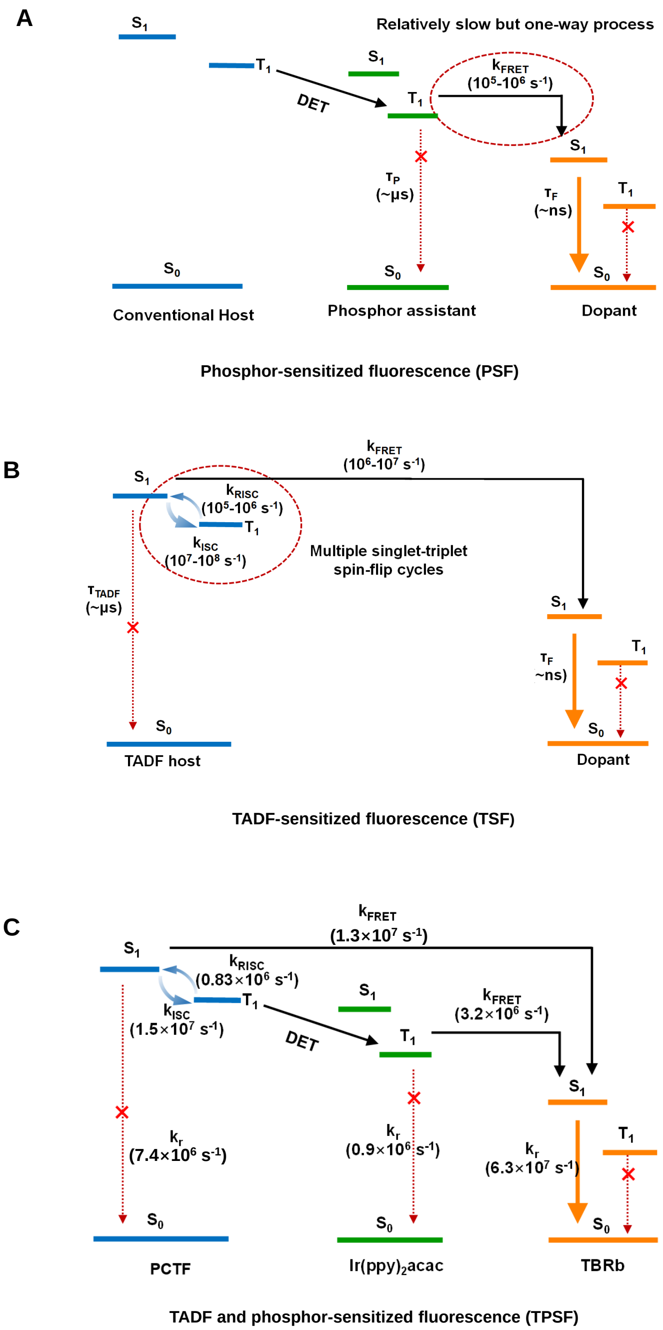

Fig. S1.

Schematic diagrams of exciton dynamics in different sensitizing mechanism. (**A**) Phosphor-sensitized fluorescence. (**B**) TADF-sensitized fluorescence. (**C**) Phosphor-assisted TADF-sensitized fluorescence (TPSF) mechanism with specific dynamic rates.

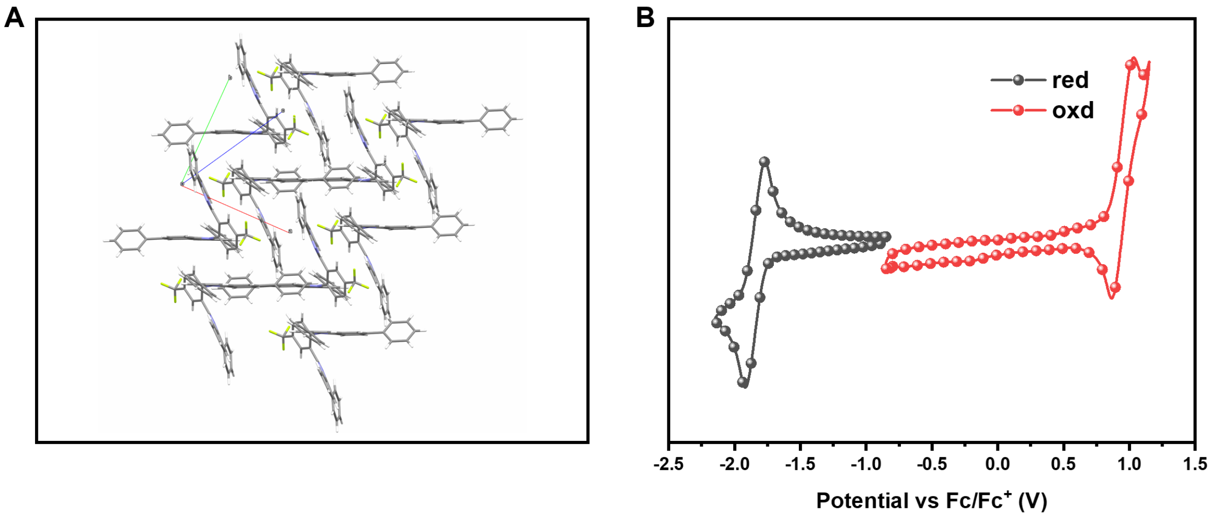

Fig. S2.

Characterization of PCTF. (**A**) Molecular packing of PCTF (CCDC No. 2128150) in single crystal. (**B**) Cyclic voltammetry spectra (oxidation and reduction) of PCTF.

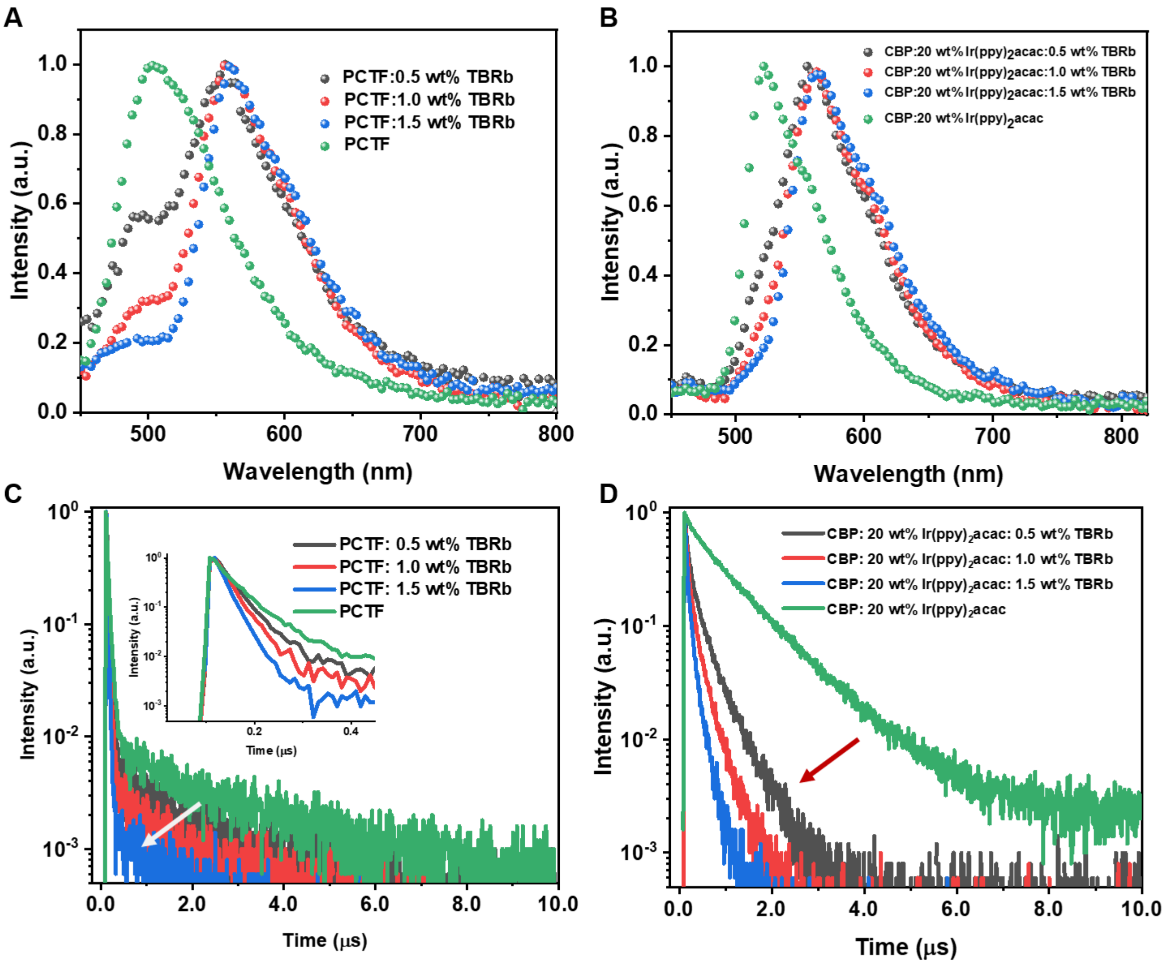

Fig. S3.

Photophysical properties of TSF and PSF vapored films with varied TBRb-doping. Photoluminescence spectra of (**A**) TSF films and (**B**) PSF films. Photoluminescence transient decay curves of (**C**) TSF films measured at 500 nm and (**D**) PSF films measured at 520 nm.

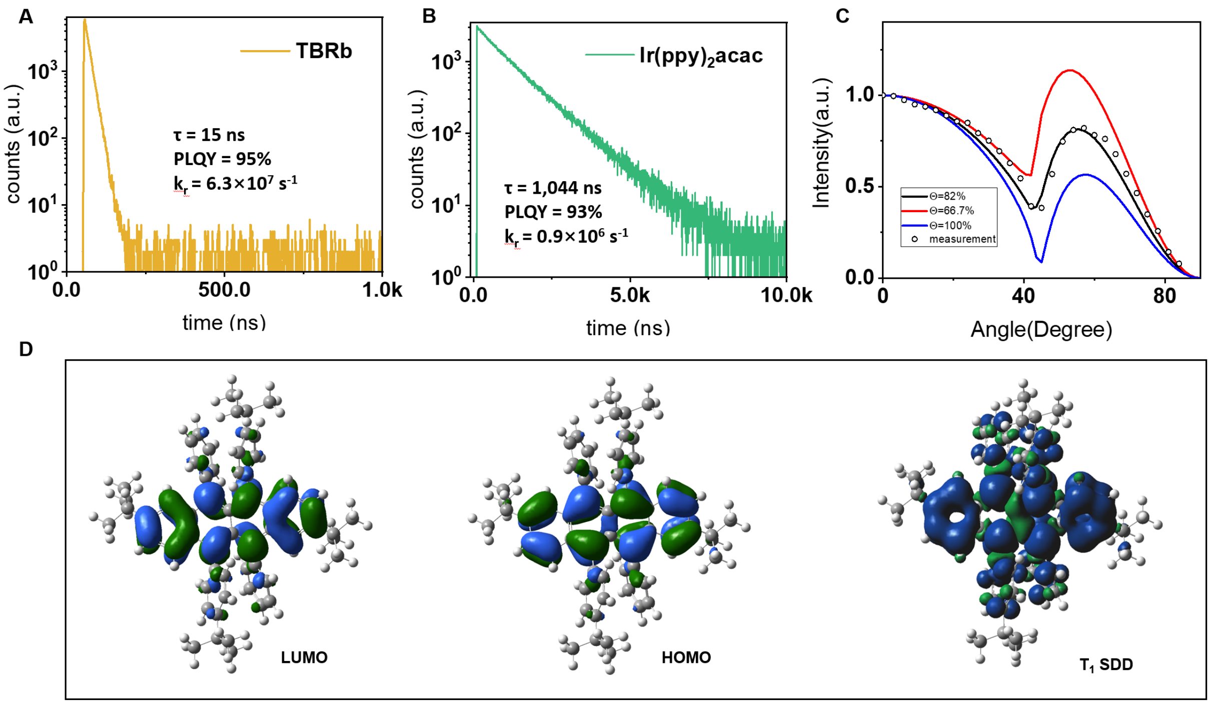

Fig. S4.

Additional characterizations of TBRb and Ir(ppy)$_2$acac. Photoluminescence transient decay curves of (**A**) TBRb and (**B**) Ir(ppy)$_2$acac in degassed toluene solution with 10^{-5} M measured at 298 K. (**C**) Angle-dependent photoluminescence intensity of *p*-polarized light of a 25-nm-thick evaporated PCTF: 20 wt% Ir(ppy)$_2$acac: 1 wt% TBRb film measured at 650 nm. (**D**) Calculated molecular orbital distribution of LUMO, HOMO and T_1 spin density distribution of TBRb.

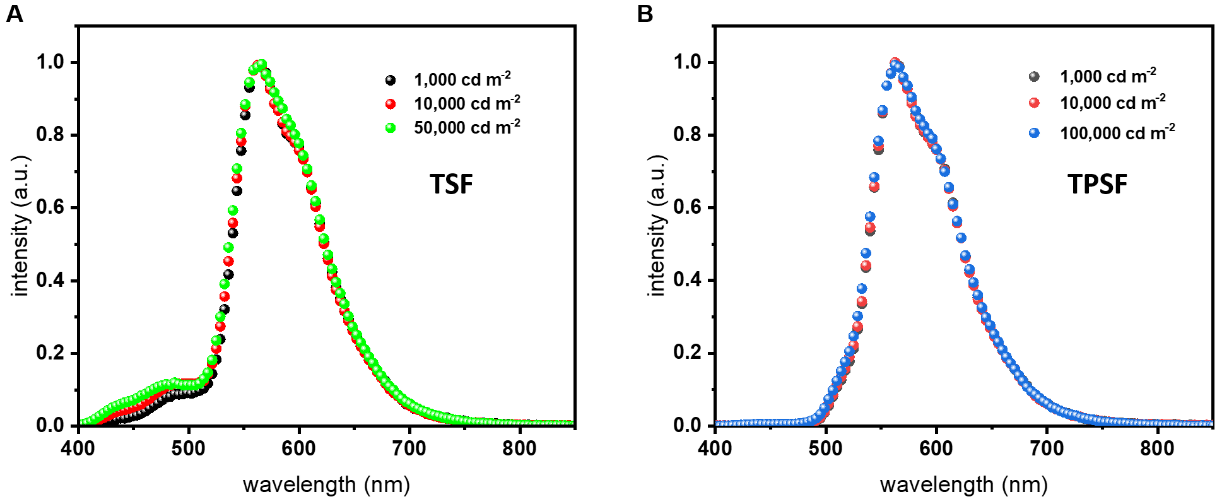

Fig. S5.

Electroluminescence spectra of TSF and TPSF devices measured at different luminance. (**A**) TSF (PCTF: 1 wt% TBRb) device. (**B**) TPSF (PCTF: 20 wt% Ir(ppy)$_2$acac: 1 wt% TBRb) device

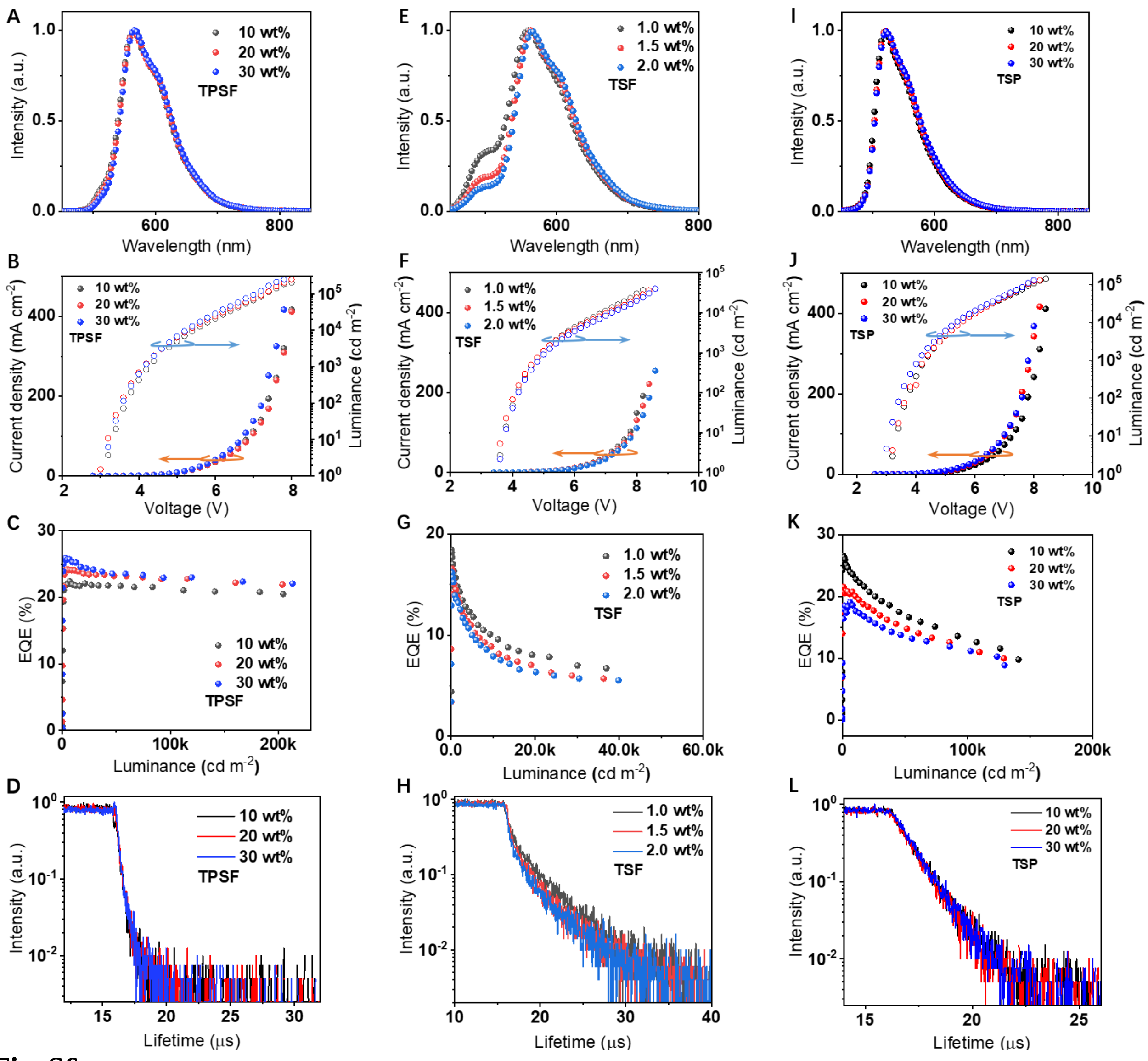

Fig. S6.

Device performances of optimized devices based on PCTF host. Specific spectra at 10,000 cd m^{-2} of (**A**) TPSF, (**E**) TSF and (**I**) TSP devices. J-V-L curves of (**B**) TPSF, (**F**) TSF and (**J**) TSP devices. EQE-luminance curves of (**C**) TPSF, (**G**) TSF and (**K**) TSP devices. Electroluminescence transient decay curves of (**D**) TPSF, (**H**) TSF and (**L**) TSP devices.

(TPSF indicating PCTF: x wt% Ir(ppy)$_2$acac: 1 wt% TBRb, TSF indicating PCTF: x wt% TBRb and TSP indicating PCTF: x wt% Ir(ppy)$_2$acac.)

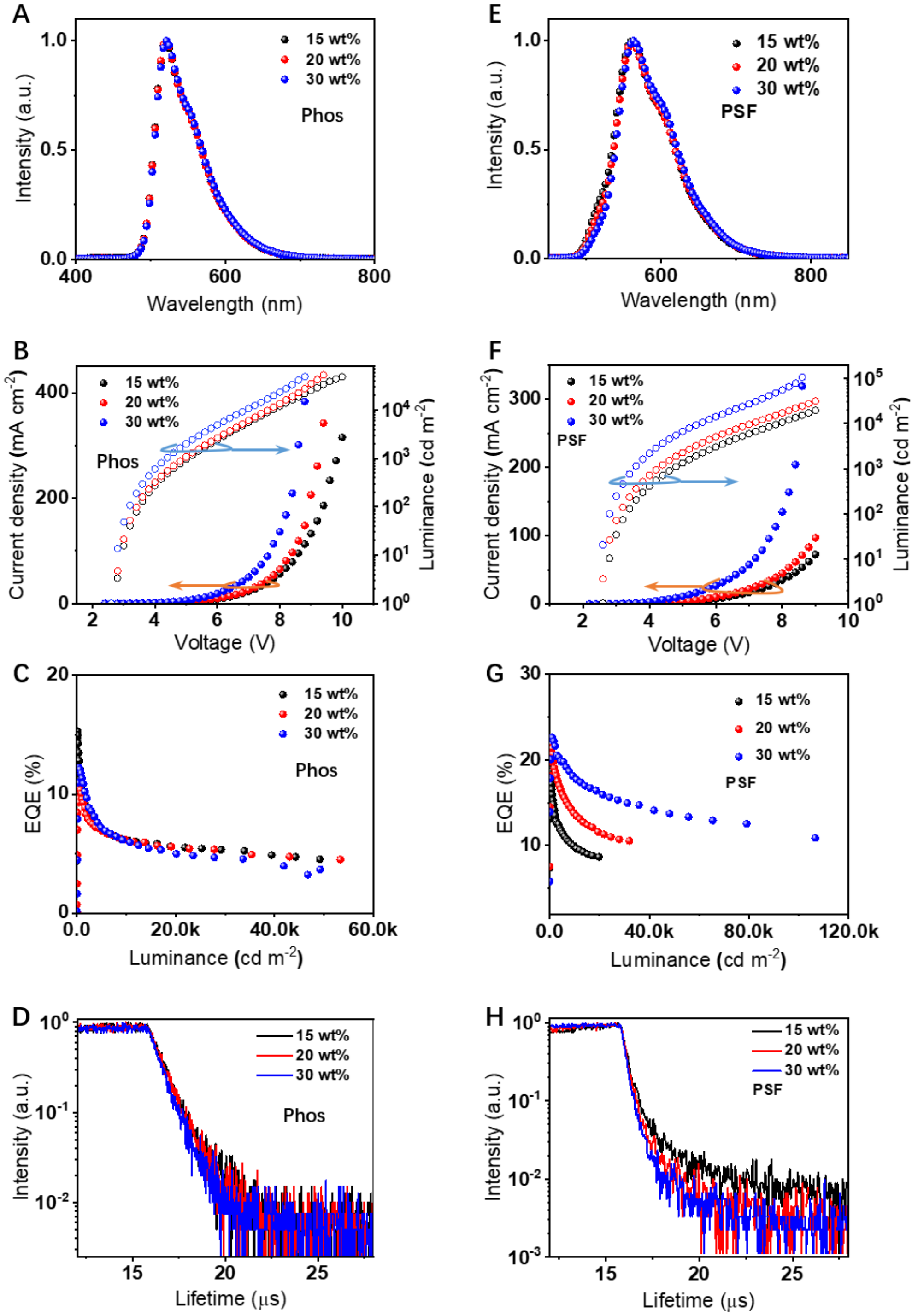

Fig. S7.
Device performances of optimized devices based on CBP host. Specific spectra at 10,000 cd m^{-2} of (**A**) Phos and (**E**) PSF devices. J-V-L curves of (**B**) Phos and (**F**) PSF devices. EQE-luminance curves of (**C**) Phos and (**G**) PSF devices. Electroluminescence transient decay curves of (**D**) Phos and (**H**) PSF devices.

(Phos indicating CBP: x wt% Ir(ppy)$_2$acac, PSF indicating CBP: x wt% Ir(ppy)$_2$acac: 1 wt% TBRb.)

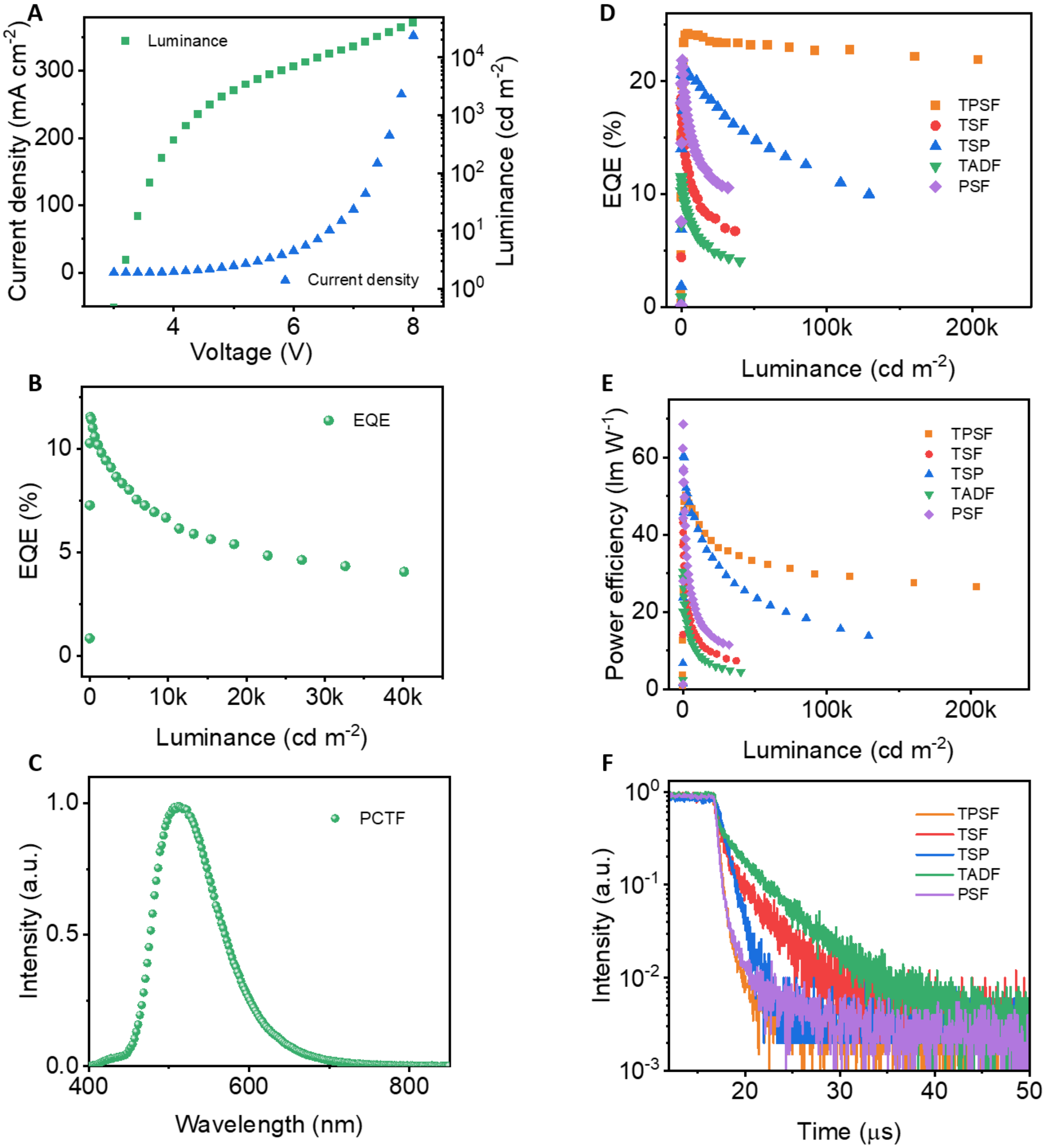

Fig. S8.

Additional device performances. (**A**) J-V-L curves, (**B**) EQE-luminance curve and (**C**) Electroluminescence spectra at 10,000 cd m^{-2} of comparison device employing only PCTF as emitting layer. (**E**) EQE-luminance curves, (**E**) Power-efficiency-luminance curves and (**E**) Electroluminescence transient decay curves of partial devices.

(TPSF indicating PCTF: 20 wt% Ir(ppy)$_2$acac: 1 wt% TBRb, TSF indicating PCTF: 1 wt% TBRb, TSP indicating PCTF: 20 wt% Ir(ppy)$_2$acac, TSF indicating pristine PCTF, PSF indicating CBP: x wt% Ir(ppy)$_2$acac: 1 wt% TBRb.)

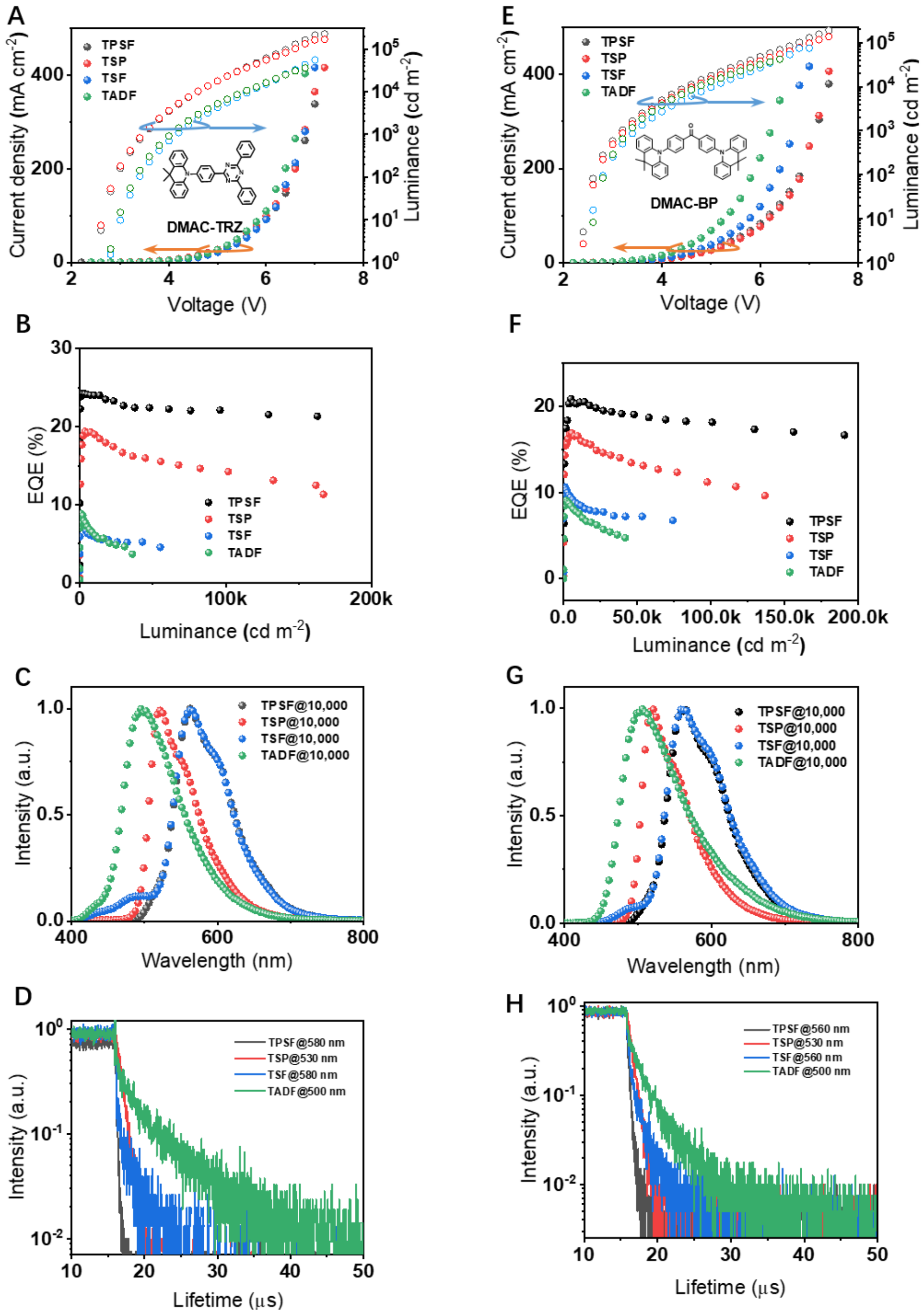

Fig. S9.
Device performances of comparison devices with specific emitting layers based on DMAC-TRZ and DMAC-BP, respectively. (**A**) Specific spectra at 10,000 cd m^{-2}. (**B**) J-V-L curves. (**C**) EQE-luminance curves. (**D**) Electroluminescence transient decay curves of comparison devices based on DMAC-TRZ. (**E**) Specific spectra at 10,000 cd m^{-2}. (**F**) J-V-L curves. (**G**) EQE-luminance curves. (**H**) Electroluminescence transient decay curves of comparison devices based on DMAC-BP.

(TPSF device was composed of TADF: 20 wt% Ir(ppy)$_2$acac: 1 wt% TBRb, TSP of TADF: 20 wt% Ir(ppy)$_2$acac, TSF of TADF: 1 wt% TBRb and TADF of pristine TADF.)

Table S1.

Summary of reported OLED devices cited in Fig. 1A.

Reference	EQE_{max} (%)	$L_{90\%}$[&] (cd m^{-2})	L_{max} (cd m^{-2})	EL peak[%] (nm)
4	18	~2,000	~10,000*	560
5	6.9	~2,000	166,000	555
6	26.1	~12,000	19,000	560
7	8.6	~3,800	52,000	560
8	19	~6,000	~48,000*	560
9	5.11	~11,000	54,300	448
10	3.9	~8,900	~400,000*	480
11	19.2	~2,000	96,000	530
12	20.6	~10,000	242,000	523
13	26.1	~3,700	32,000	570
14	25.1	~6,000	93,330	520
15	23.85	~4,000	114,900	568
16	22	~34,000	~100,000*	520
17	24	~2,000	~70,000*	520
18	17.2	~15,000	30,000*	520
19	15.6	~4,000	74,280	520
20	19.2	~3,000	69,190	500
21	31.6	~3,000	71,160	526
22	37.8	~2,200	73,000	528
23	38.15	~1,200	47,680	480

& Estimated based on roll-off figures;
**** Estimated based on L-V or L-J figures;***
% Estimated based on electroluminescence spectra.

Table S2.

Summary of dynamic rates of TSF and PSF as depicted in Fig. S3.

Doping of TBRb	TSF (measured at 490 nm)		PSF (measured at 520 nm)	
	τ_P (ns)	k_{FRET}^{*} (10^6 s^{-1})	τ_P (ns)	$k_{ET}^{\&}$ (10^6 s^{-1})
0	41.2		664	
0.5 wt%	38.1	2.03	344	1.41
1.0 wt%	26.6	13.4	212	3.20
1.5 wt%	20.9	23.5	126	6.41

$^{*}k_{FRET} \approx k_P - k_{ISC} - k_{nr}$
$^{\&}k_{ET} \approx k_P - k_{nr}$
$k_P = 1/\tau_P$

Table S3.
Performance summary of all optimized and comparison devices in Supplementary Material.

device	Doping/ type	EQE_{max}	EQE (at 10,000 cd m^{-2})	EQE (at 50,000 cd m^{-2})	EQE (at 100,000 cd m^{-2})	$L_{90\%}$ (cd m^{-2})
TPSF	10 wt%	22.4%	21.8%	21.7%	21.2%	248,000
	20 wt%	24.2%	24.0%	23.2%	22.7%	190,500
	30 wt%	25.5%	25.2%	23.5%	22.9%	120,000
TSF	1.0 wt%	18.4%	9.7%	null	null	720
	1.5 wt%	16.5%	8.5%	null	null	860
	2.0 wt%	16.2%	7.9%	null	null	800
TSP	10 wt%	26.5%	22.8%	17.5%	12.7%	8,400
	20 wt%	21.6%	20.1%	14.8%	11.1%	13,280
	30 wt%	19.0%	17.8%	13.5%	11.2%	16,000
TADF	PCTF	11.6%	6.6%	null	null	890
Phos	15 wt%	15.3%	6.2%	4.5%	null	400
	20 wt%	12.6%	6.0%	4.5%	null	1,300
	30 wt%	12.2%	5.9%	3.1%	null	1,600
PSF	15 wt%	18.8%	9.8%	null	null	900
	20 wt%	21.9%	13.4%	null	null	1,600
	30 wt%	22.6%	17.7%	13.6%	12.9%	3,700
DMAC -TRZ	TPSF	24.8%	24.0%	22.4%	22.0%	129,000
	TSP	19.4%	19.0%	15.7%	14.3%	23,000
	TSF	8.0%	5.8%	4.7%	null	3,100
	TADF	8.8%	6.0%	null	null	2,900
DMAC -BP	TPSF	20.2%	20.0%	19.0%	18.2%	101,000
	TSP	16.8%	16.1%	13.2%	11.0%	22,000
	TSF	10.6%	8.5%	7.2%	null	4,500
	TADF	9.0%	7.6%	null	null	6,600
